# Case Report: Epstein-Barr-Virus negative diffuse large B-cell lymphoma detected in a peri-prosthetic membrane 

**DOI:** 10.1186/s13000-016-0533-z

**Published:** 2016-08-18

**Authors:** Sandra Sunitsch, Magdalena Gilg, Karl Kashofer, Andreas Leithner, Bernadette Liegl-Atzwanger, Christine Beham-Schmid

**Affiliations:** 1 Institute of Pathology, Medical University of Graz, Graz, Austria; 2 Department of Orthopedics and Orthopedic Surgery, Medical University of Graz, Graz, Austria

**Keywords:** Primary bone lymphoma, Diffuse large B-cell lymphoma, Epstein-Barr-Virus negative, Knee arthroplasty, Peri-prosthetic membrane

## Abstract

**Background:**

Primary bone lymphomas (PBL) are extremely rare malignant neoplasms. The most commonly described subtype of PBL is diffuse large B-cell lymphoma (DLBCL). DLBCL within peri-prosthetic membrane of a joint is exceedingly rare. To the best of our knowledge this case is the second reported Epstein-Bar-Virus (EBV) negative DLBCL in a peri-prosthetic membrane in the literature.

**Case presentation:**

We report an 80 year old female patient who developed a DLBCL with chronic inflammation in association to a metallic implant in the left knee. This lymphoma in contrast to the usually described DLBCL in the peri-prosthetic membrane was EBV negative by EBER in situ hybridization as well as by polymerase chain reaction (PCR).

**Conclusion:**

This report challenges the concept of DLBCL associated with chronic inflammation and raises the question of other pathogenetic factors involved in the pathogenesis of this rare disease.

## Background

Lymphoma is the seventh most common systemic malignancy worldwide [1, 2]. DLBCL is the most common aggressive Non-Hodgkin Lymphoma (NHL) [3] and in addition the most common type of PBL [4]. Over the last decades, the use of joint replacement surgery, such as total hip and total knee arthroplasty, has increased [2]. Despite this fact lymphomas developed within the peri-prosthetic membrane are exceedingly rare. These lymphomas may be observed in a context of chronic inflammation with EBV, which seems to play a central role in disease development. Since 2008 this entity has been included in the World Health Organization (WHO) classification of Haematopoietic and Lymphoid Tissues as “DLBCL associated with chronic inflammation” [1, 5].

**Fig. 1:**
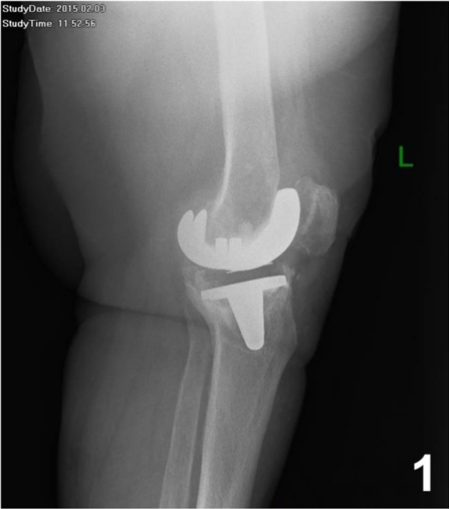
Radiography of the left knee shows a loosening of the prosthesis

**Fig. 2:**
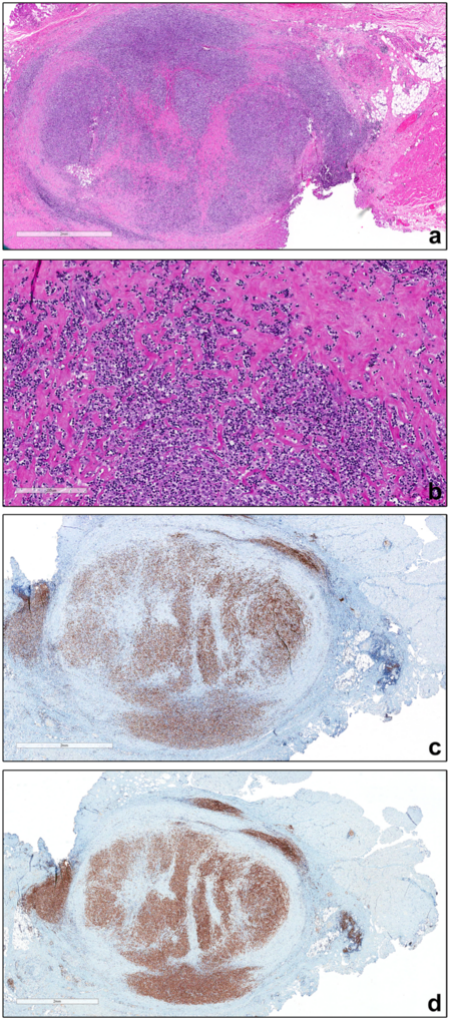
**a** HE stained section shows connective tissue with massive hyalinization and sclerosis and skeletal muscle with a centrally, circumscribed, nodular infiltration of large lymphoid cells. **b** The predominant cellular component consisted of blasts, with round and notched nuclei with clear chromatin and multiple nucleoli. **c** In the immunohistochemistry staining the blasts reveal an expression of CD 79a. **d** In the immunohistochemistry staining the blasts reveal an expression of CD 20

## Case presentation

In 2004 a 69 year old woman underwent total left knee replacement with a cemented knee endoprosthesis for osteoarthritis. In February 2015 a loosening of the prosthesis was diagnosed radiologically (Fig. 1) in association with a rupture of the collateral ligament.

The patient underwent replacement of the prosthesis in August 2015 with ASA 4 (American Society of Anesthesiology Classification) because of multiple comorbidities. The listed comorbidities included adipositas, diabetes mellitus, high blood pressure, hyperlipidaemia and coronary heart disease. In addition the patient showed in clinical chemistry positivity for HBVc (Hepatitis B Virus core) antibody but negativity for Hepatitis B by PCR.

The peri-prosthetic membrane was removed and a new prosthesis was implanted without complications. The peri-prosthetic membrane was sent to the Institute of Pathology to exclude infection. The histological examination showed infiltration by a DLBCL. Clinically the patient showed no signs of generalization.

A chemotherapy with (R-)-mini-CHOP (with 50 % of Cyclophospamid, Doxorubicin and Oncovin) was started. Because of the positivity of HBVc antibody the Mabthera therapy started in the 2nd cycle after confirmation of negative Hepatitis B PCR. After chemotherapy she received a total irradiation dose 40 Gy with conventional fractions.

At a follow-up of 8 months the patient did not show any sign of recurrence.

## Results

The specimen sent for histological examination measured 2:1,5:0,8 cm and revealed grey-white tissue.

The histolologic examination showed connective tissue with massive hyalinization and sclerosis with a centrally, circumscribed, nodular infiltration of large lymphoid cells (Fig. 2a). The predominant cellular component consisted of blasts, with round and notched nuclei with clear chromatin and multiple nucleoli (Fig. 2b). The mitotic activity was high with 4 mitoses per high power field. Between the blasts a few polymorphic small reactive lymphocytes were present. The blasts showed strong expression of CD 20, CD 79a (Fig. 2c), CD20 (Fig. 2d), PAX 5 and bcl-2 by immunohistochemistry.

Only a small subset of blasts demonstrated a positive staining with CD 30 and bcl-6 (<5 %). Staining for EBER (in situ hybridisation for EBV with INFORM EBER) and CD 15 were negative. In addition the atypical B-cell population did not express CD10, MUM1, GCET1, CD33, myeloperoxidase, C-MYC, CD117c and FOXP1. The proliferation index (MIB) was 80 %.

The diagnosis of a DLBCL was supported by performing a PCR, demonstrating a gene rearrangement in the immunoglobulin heavy chain gene with a monoclonal peak at 243 bp and an immunoglobulin light chain gene rearrangement with a monoclonal cell population kappa A at 288 bp.

EBV was additionally excluded by a PCR using Lightcycler analysis.

Performing a Low Density Whole Genome Copy Number Variation with next generation sequencing (NGS) we could not detect any copy number variation. Furthermore mutational analysis of the full coding sequence of the TP 53 gene did not demonstrate mutations in TP 53.

## Discussion

Primary lymphoma of the bone is rare, accounting for 3–7 % of malignant bone neoplasms and 4–5 % of extra-nodal Non-Hodgkin lymphomas [6]. Primary Non-Hodgkin lymphomas arising in the peri-prosthetic membrane around metallic implants are extremely rare. The carcinogenic effects of the metallic components and wear particles are not yet fully elucidated [7]. Most cases reported in this context are DLBCL associated with chronic inflammation and concomitant EBV infection [2, 7]. Campo et al. report, that in these lesions lymphomas-infected cells frequently express a latency type 3 with positivity for the viral proteins latent membrane protein 1 and EBV nuclear antigen 2, suggesting, that the lymphoid proliferation may be related primarily to a decrease in the host immune-surveillance [5]. Our described case of DLBCL, however, lacks EBV infection and is localized to the knee without any other site of involvement. A summary of our literature search (see Table 1) showed only one case of diffuse large B-cell lymphoma in association with a joint replacement without EBV association and was published in 2011 [2]. Another case was reported with negativity for the EBV in situ hybridization, but positivity for EBV in the polymerase chain reaction [7]. All reported cases did not show any signs of generalization [2, 7–13].

**Table 1:** Shows a summary of presented cases in the literature including our recent case

Journal	Age/Sex	Localisation	Latency	Diagnosis	EBV status
Our case	80 female	Left knee	11 years	DLBCL	EBERinsitu -PCR -
Acta Haematol. 2011;126(3):141–6. [2]	76 male	Right Tibia	3 years	DLBCL	EBERinsitu -
Am J Surg Pathol 2005; 29: 832–836. [8]	78 male	Right knee	22 years	DLBCL	EBV IHC + EBERinsitu +
Journal of Clinical Oncology, Vol 31, 2013: pp e148-e151. [7]	69 male	Left knee	3 years	DLBCL	EBERinsitu - PCR +
Arch Orthop Trauma Surg 2008; 128: 1387–1390. [9]	70 female	Left knee	About a half year	DLBCL	not available
J Arthroplasty. 2001 Feb;16(2):229–32. [10]	97 female	Left hip	12 years	High-grade Non-Hodgkin’s B-cell lymphoma	not available
J Arthroplasty. 2006 Sep;21(6):926–30. [11]	75 female	Right tigh	13 years	Malignant immunoblastic lymphoma	not available
J Clin Pathol. 1998 Aug; 51(8): 629–632. [12]	63 female	Left tigh	4 years	Diffuse centroblastic lymphoma	not available
	25 male	Right tibia	8 years	Diffuse centroblastic lymphoma	not available
Cancer. 1981 Aug 15;48(4):1009–11. [13]	48 male	Left tibia	17 years	Malignant lymphoma	not available

In the context of our case we would like to challenge the concept that “DLBCL associated with chronic inflammation” is always associated with EBV infection. In our case EBV could clearly not be demonstrated by otherwise classic features of “DLBCL associated with chronic inflammation”. Although the pathogenesis in our case is not fully elucidated, we hypothesize that other factors may play a role in this context. Especially metallic ions are known to have carcinogenic potential. After a metal implantation a significant increased concentration of cobalt and chromium in the synovial fluid and blood could be measured. These metallic ions have mutagenic effects and have been described to potentially predispose to lymphomas [2]. In 2004 Landon et al. report about an increase in chromium and cobalt levels, an increased incidence of chromosomal translocation and an aneuploidy in the peripheral blood of patients within 2 years after metal-on-metal joint arthroplasty [14].

In addition a higher level of HBVc antibody was incidentally found in our patient. Gross et al. states that a negative HBV DNA does not rule out an HBV infection [15].

In the literature a connection between Hepatitis B virus infection and Non-Hodgkin lymphomas is described [16–21]. It is suggested that HBV is not only a hepatotropic but also a lymphotropic disease and participates in the development of malignant lymphoproliferative disorders. For viral clearance the cellular and humoral immunity plays an important role for HBV infection and disease progression. The cellular and humoral immunity system gets activated after HBV infection. The immune system destroys the HBV infected cells and the host cells, which are infected by HBV [18]. However, the mechanisms are not fully elucidated [19, 20] and several theories are suggested. Proposed theories include [19]:

- Chronic stimulation of B-cells is the problem for DNA damage and transformation into B- cell NHL.
- Chronic local antigenic stimulation could be associated with a development of lymphoma.
- HBV-encoded X-protein (HBx) inhibits p53 in hepatocytes and this mechanism causes hepatocellular carcinoma. The inhibition of p53 in B-cells may cause malignant transformation into a NHL. However this theory can be excluded by performing mutational analysis covering the whole coding sequence of TP53.
- The infection of epithelial cells might serve as a trigger for expression, production or release of hematopoietic tumor growth factor.
- A coinfection with another unknown virus is suggested to cause lymphoma.

## Conclusion

We report a case of an 80 year old woman, who developed an EBV negative DLBCL in the peri-prosthetic membrane 11 years after total knee arthroplasty. The presented case demonstrates that EBV infection might not be obligatory in DLBCL associated with chronic inflammation. This report challenges the concept of DLBCL associated with chronic inflammation and raises the question of other pathogenetic factors involved in the pathogenesis of this rare disease.

## Abbreviations

ASA: American Society of Anesthesiology Classification
DLBCL: Diffuse large B-cell lymphoma
EBV: Epstein- Bar-Virus
Gy: Gray
HBV DNA: Hepatitis B Virus Deoxyribonucleic acid
HBVc: Hepatitis B Virus core
HBx: HBV-encoded X-protein
NGS: Next generation sequencing
NHL: Non-Hodgkin Lymphoma
PBL: Primary bone lymphoma
PCR: Polymerase chain reaction
WHO: World Health Organization
